# Identification of *C. elegans *RAB-5-dependent downstream effectors as regulators of ciliogenesis and ciliary membrane homeostasis

**DOI:** 10.1186/2046-2530-4-S1-P77

**Published:** 2015-07-13

**Authors:** N Scheidel, O Blacque

**Affiliations:** 1 University College Dublin, Dublin, Ireland

## 

Cilia are microtubule based organelles enveloped by a specialised extension of plasma membrane. Serving as cellular antennae, cilia are essential for perceiving extracellular cues and mediating downstream signal transduction crucial for normal cellular physiology and development. These functions are mediated in large part by the specialised ciliary membrane, which differs in composition from the plasma membrane, housing many of the receptors, channels and effectors that mediate cilium-based signalling. However, the molecular mechanisms underlying the establishment, maintenance and regulation of the ciliary membrane remain poorly understood.

In this project we are seeking to identify and functionally characterize genes with ciliogenic roles in ciliary membrane transport and organisation. Specifically, we are taking a candidate gene approach using the *Caenorhabditis elegans *ciliated sensory neuronal model, employing assays for cilium integrity (dye filling assay) and functionality (osmotic avoidance assay; foraging).

Using predicted loss-of-function alleles, we screened 55 mutants of evolutionarily conserved membrane organising and trafficking genes and identified 4 *rab-5*-related endocytic gene mutants with defects in cilium integrity and function. One of these, *rabs-5*, encodes a RAB-5 effector, which we found is expressed mainly in ciliated cells. *rabs-5 *mutants possess short cilia and an expanded periciliary membrane compartment, indicative of defects in ciliary membrane homeostasis. Transgenic expression of a *rabs-5(WT) *construct rescues the cilium integrity and foraging defects of *rabs-5 *mutants, confirming the association of *rabs-5 *with cilia-related functions.

By taking a reverse genetics candidate gene approach in *C. elegans*, a number of RAB-5-dependent downstream effectors have been identified as potential regulators of ciliogenesis and ciliary membrane homeostasis.

